# Supplementary material for: Cystic Fibrosis in Adults: A Paradigm of Frailty Syndrome? An Observational Study
Source: J Clin Med. 2024 Jan 19;13(2):585. doi: 10.3390/jcm13020585 (PMC10816671; doi:10.3390/jcm13020585)
Supplement: Supplementary file 1 [file jcm-13-00585-s001.zip › jcm-2744686-supplementary.pdf]

SUPPLEMENTAL MATERIAL

Table S1. Post hoc power analysis

(A)

```
Estimated power for a two-sample means test
Satterthwaite's t test  assuming  unequal  variances
Ho: m2  = m1  versus  Ha: m2  != m1

Study   parameters:

          alpha  =      0.0500
              N  =         139
             N1  =          84
             N2  =          55
          N2/N1  =      0.6548
          delta  =     -1.7300
             m1  =      3.2800
             m2  =      1.5500
            sd1  =      0.9000
            sd2  =      0.7000

Estimated   power:

         power  =     1.0000
```

(B)

```
Estimated  sample  sizes  for  a  two-sample  means  test
Satterthwaite's t test  assuming  unequal  variances
Ho: m2 = m1 versus  Ha: m2  != m1

Study  parameters:

         alpha  =     0.0500
         power  =     0.8000
         delta  =    -1.7300
            m1  =     3.2800
            m2  =     1.5500
           sd1  =     0.9800
           sd2  =     0.7500

Estimated  sample  sizes:

             N =          12
  N per group =            6
```

(C)

```
Estimated  sample  sizes  for  a  two-sample  means  test
Satterthwaite's t  test  assuming  unequal  variances
Ho: m2  = m1  versus  Ha: m2  != m1

Study   parameters:

         alpha  =      0.0500
         power  =      0.9000
         delta  =     -1.7300
            m1  =      3.2800
            m2  =      1.5500
           sd1  =      0.9800
           sd2  =      0.7500

Estimated   sample   sizes:

             N  =          14
  N per group  =            7
```

Table S2. Main characteristics of the study population

Variables	Total Population (n=139)
Age (yrs), mean±SD (range)	32.89±10.94 (18-67)
Gender (M/F), n° (%)	75/64 (54/46)
BMI (kg/m^2), mean±SD (range)	23.93±4.01 (16.6-39.0)
FEV_1 (L), mean±SD	2.60±1.24
FEV_1%, mean±SD	69.91±29.87
FVC (L), mean±SD	3.54±1.34
FVC%, mean±SD	81.80±24.20
MMEF%, mean±SD	56.55±37.69
n° pulmonary exacerbations per year, mean±SD	1.37±2.02
Cycles of antibiotic IV therapy, mean±SD	0.28±0.89
Cycles of antibiotic oral therapy, mean±SD	1.06±1.54
n° hospitalizations per year, mean±SD	0.25±0.64
BADL (lost), mean±SD	0.09±0.49
IADL (lost), mean±SD	0.26±1.18
n° drugs, mean±SD	6.12±4.06
n° CF-related diseases, mean±SD	3.85±2.98
n° diseases non-CF-related, mean±SD	0.37±0.69
n° total diseases, mean±SD	4.22±3.06
Smokers (yes), n (%)	7 (5)
**Main Genotypes**	
DeltaF508/DeltaF508, n (%)	27 (19.4)
DeltaF508/5T-12TG, n (%)	11 (7.9)
Delta F508/N1303K, n (%)	5 (3.6)
**Frailty phenotype**	
Robust, n° (%)	84 (60.4)
Pre-frail/Frail, n° (%)	55 (39.6)
**Main Infection agents**	
Staphiylococcus Aureus (yes), n° (%)	125 (89.9)
Pseudomonas Aeruginosa (yes), n° (%)	73 (52.5)
Achromobacter (yes), n° (%)	13 (9.4)
Stenotrophomonas (yes), n° (%)	3 (2.2)
Klebsiella (yes), n° (%)	3 (2.2)
Mycobacterium abscessus (yes), n° (%)	3 (2.2)
Acinetobacter (yes), n° (%)	3 (2.2)
Burkholderia cepacia complex (yes), n° (%)	2 (1.4)

STROBE Statement—checklist of items that should be included in reports of observational studies

	Item No.	Recommendation	Page No.
**Title and abstract**	1	(*a*) Indicate the study's design with a commonly used term in the title or the abstract	1, 2
		(*b*) Provide in the abstract an informative and balanced summary of what was done and what was found	2
**Introduction**			
Background/rationale	2	Explain the scientific background and rationale for the investigation being reported	3
Objectives	3	State specific objectives, including any prespecified hypotheses	4
**Methods**			
Study design	4	Present key elements of study design early in the paper	4
Setting	5	Describe the setting, locations, and relevant dates, including periods of recruitment, exposure, follow-up, and data collection	4
Participants	6	(*a*) *Cross-sectional study*—Give the eligibility criteria, and the sources and methods of selection of participants	4
		(*b*) *Cohort study*—For matched studies, give matching criteria and number of exposed and unexposed<br>*Case-control study*—For matched studies, give matching criteria and the number of controls per case	NA
Variables	7	Clearly define all outcomes, exposures, predictors, potential confounders, and effect modifiers. Give diagnostic criteria, if applicable	5
Data sources/ measurement	8*	For each variable of interest, give sources of data and details of methods of assessment (measurement). Describe comparability of assessment methods if there is more than one group	*5*
Bias	9	Describe any efforts to address potential sources of bias	5,6
Study size	10	Explain how the study size was arrived at	5
Quantitative variables	11	Explain how quantitative variables were handled in the analyses. If applicable, describe which groupings were chosen and why	5
Statistical methods	12	(*a*) Describe all statistical methods, including those used to control for confounding	5
		(*b*) Describe any methods used to examine subgroups and interactions	5
		(*c*) Explain how missing data were addressed	5
		(*d*) *Cross-sectional study*—If applicable, describe analytical methods taking account of sampling strategy	5
**Results**			
Participants	13*	(a) Report numbers of individuals at each stage of study—eg numbers potentially eligible, examined for eligibility, confirmed eligible, included in the study, completing follow-up, and analysed	6, Table S2
		(b) Give reasons for non-participation at each stage	NA

		(c) Consider use of a flow diagram	NA
Descriptive data	14	(a) Give characteristics of study participants (eg demographic, clinical, social) and information on exposures and potential confounders	6-12<br>Tables 1-3
		(b) Indicate number of participants with data for each variable of interest	6-12
		(c) *Cohort study*—Summarise follow-up time (eg, average and total amount)	NA
Outcome data	15	*Cross-sectional study*—Report numbers of outcome events or summary measures	Table 1-4,<br>Figs 1,2,<br>Table S2
Main results	16	(*a*) Give unadjusted estimates and, if applicable, confounder-adjusted estimates and their precision (eg, 95% confidence interval). Make clear which confounders were adjusted for and why they were included	Table 4
		(*b*) Report category boundaries when continuous variables were categorized	6-12
		(*c*) If relevant, consider translating estimates of relative risk into absolute risk for a meaningful time period	7, Table 4

Other analyses	17	Report other analyses done—eg analyses of subgroups and interactions, and sensitivity analyses	Table 4,<br>Supplemental material
**Discussion**			
Key results	18	Summarise key results with reference to study objectives	12-14
Limitations	19	Discuss limitations of the study, taking into account sources of potential bias or imprecision. Discuss both direction and magnitude of any potential bias	14,15
Interpretation	20	Give a cautious overall interpretation of results considering objectives, limitations, multiplicity of analyses, results from similar studies, and other relevant evidence	15
Generalisability	21	Discuss the generalisability (external validity) of the study results	15
**Other information**			
Funding	22	Give the source of funding and the role of the funders for the present study and, if applicable, for the original study on which the present article is based	15
